# Proteomic patterns of cervical cancer cell lines, a network perspective

**DOI:** 10.1186/1752-0509-5-96

**Published:** 2011-06-22

**Authors:** Juan Carlos Higareda-Almaraz, María del Rocío Enríquez-Gasca, Magdalena Hernández-Ortiz, Osbaldo Resendis-Antonio, Sergio Encarnación-Guevara

**Affiliations:** 1 Centro de Ciencias Genómicas, Universidad Nacional Autónoma de México. Apdo, Postal 565-A, Cuernavaca, Morelos, CP 62210, México; 2 Licenciatura en Ciencias Genómicas, Universidad Nacional Autónoma de México. Apdo. Postal 565-A, Cuernavaca, Morelos, CP 62210, México

## Abstract

**Background:**

Cervical cancer is a major mortality factor in the female population. This neoplastic is an excellent model for studying the mechanisms involved in cancer maintenance, because the Human Papilloma Virus (HPV) is the etiology factor in most cases. With the purpose of characterizing the effects of malignant transformation in cellular activity, proteomic studies constitute a reliable way to monitor the biological alterations induced by this disease. In this contextual scheme, a systemic description that enables the identification of the common events between cell lines of different origins, is required to distinguish the essence of carcinogenesis.

**Results:**

With this study, we sought to achieve a systemic perspective of the common proteomic profile of six cervical cancer cell lines, both positive and negative for HPV, and which differ from the profile corresponding to the non-tumourgenic cell line, HaCaT. Our objectives were to identify common cellular events participating in cancer maintenance, as well as the establishment of a pipeline to work with proteomic-derived results. We analyzed by means of 2D SDS-PAGE and MALDI-TOF mass spectrometry the protein extracts of six cervical cancer cell lines, from which we identified a consensus of 66 proteins. We call this group of proteins, the "central core of cervical cancer". Starting from this core set of proteins, we acquired a PPI network that pointed, through topological analysis, to some proteins that may well be playing a central role in the neoplastic process, such as 14-3-3ζ. *In silico *overrepresentation analysis of transcription factors pointed to the overexpression of c-Myc, Max and E2F1 as key transcription factors involved in orchestrating the neoplastic phenotype.

**Conclusions:**

Our findings show that there is a "central core of cervical cancer" protein expression pattern, and suggest that 14-3-3ζ is key to determine if the cell proliferates or dies. In addition, our bioinformatics analysis suggests that the neoplastic phenotype is governed by a non-canonical regulatory pathway.

## Background

The definition of cancer has evolved according to the knowledge and perspective of the scientific context in which it is conceived. It has changed from a highly heterogeneous disease seen from a cell type and tissue of origin point of view, to the conception of cancer as an illness that involves the deregulation of various pathways that govern key, and somewhat common, cellular processes [1]. Particularly, in 2000 Hanahan and Weinberg suggested that all cancer types represent a manifestation of six essential alterations in cell physiology that collectively coordinate the malignant phenotype: self-sufficiency in growth signals, insensitivity to growth inhibitors, evasion of programmed cell death, increase of the replicative potential, sustained angiogenesis and tissue invasion and metastasis [2]. Furthermore, in a recent review published by the same two authors, they proposed two emerging hallmarks: reprogramming of energy metabolism and evading immune destruction; besides suggesting genomic instability and mutations, as well as tumor-promoting inflammation, as enabling characteristics [3].

Regardless of the latter, several cancer types have been more intensively studied due to their penetrance in the human population, such as prostate and breast cancer. However, we see in cervical cancer a unique opportunity to study the malignant transformation because of its common origin: 90.7% of the cases arise as a consequence of High-Risk Human Papilloma Virus (HR-HPV) infection, according to a study carried out in nine countries of diverse cervical cancer incidences [4]. HR-HPVs encode the E6 and E7 oncoproteins, which interact with the well known and very common, tumor suppressor proteins p53 and pRB, respectively; among several other proteins that together impart a very strong oncogenic potential to the virus [5-7].

To date 120 types of human papillomaviruses have been identified, which can be subdivided into low-risk types, found mainly in genital warts, and high-risk types, which are frequently associated with invasive cervical cancer [8]. Among the high-risk types HPV16 and HPV18 are the most prevalent, present in 54.6% and 11% of squamous cervical carcinomas, respectively [4]. This is part of the reason why, cervical cancers derived from patients infected with those viral types, have been intensively studied, and one of the best studied human cell line, HeLa, is positive for HPV18 [9]. Likewise, it is pertinent to mention that there are other cell lines which originated from HPV negative cervical cancers that have also been widely studied and therefore, enable the visualization of alterations in protein expression, common to many cervical cancer cell lines independently of their origin.

Cervical cancer is one of the most common types of cancer and a major mortality factor of women worldwide [4]. Because most of the cases are a consequence of viral infections, cervical cancer is a disease that has been successfully addressed in developed countries thanks to preventive medicine [10]. Nonetheless, when left unattended, a persistent viral infection combined with a strong and constitutive expression of viral oncoproteins E6 and E7 are highly inductive steps towards the malignant transformation of cervical epithelium [5]. However, owing to a frequent and spontaneous elimination of viral sequences, not all the patients infected with HR-HPV develop cervical cancer [11]. This indicates that most HPV infections are subclinical and only a small fraction of HR-HPV infections produce early epithelial lesions, and a more modest fraction of those lesions will develop into cancer. Consequently, even if infection by HR-HPV can be considered as the initial hit that gives rise to cervical cancer, the superseding steps that enable cancer development have not yet been described [12].

Currently, an accurate prediction of the evolution of the tumor is one of the biggest challenges for clinical oncology. Because of this, the conception of an integrative model that enables the prediction of future states of a system has become of vital importance. We consider that one of the best approaches currently available to accomplish this task are protein-protein interactions (PPI), because they encompass the scaffold of molecular pathways and cellular processes, besides being capable of revealing the dynamic and interactive function of human proteins [13]. Furthermore, available databases of some organisms have promoted the construction of networks which become the starting point to explore and infer the fundamental principles by which the cell orchestrates its response to different kinds of perturbations.

In this work, we developed a pipeline for the functional analysis of differentially expressed proteins in cervical cancer compared with a non-cancerous control and obtained from 2D SDS-PAGE. We used PPI networks, pathways and GOs enrichment analysis, succeeded by an analysis of the promoter gene sequences to get a more systemic point of view of cervical cancer. We consider that the main advantage of following this approach is that it enabled to explore and locate important proteins for the biological phenomenon, which could have been overlooked as a consequence of the experimental methodology used, because some proteins are present in low concentrations or posses isoelectric points or molecular weights located outside the electrophoretic resolution range utilized in 2D SDS-PAGE.

## Results

### A conserved protein expression pattern exists in cervical cancer cell lines

**Figure 1:**
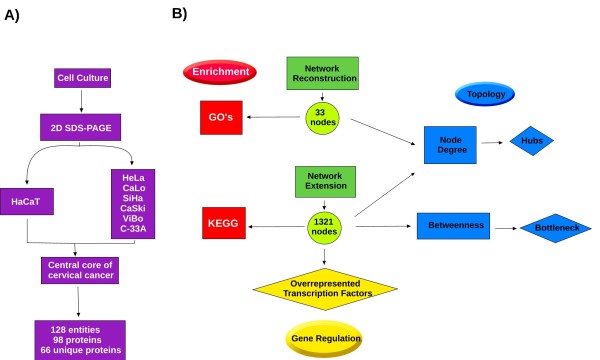
**Pipeline**. We compared the protein profiles of six cervical cancer cell lines by 2D SDS-PAGE, and established a set of proteins common to these cell lines, and which did not feature in our control. This set of proteins was termed "central core of cervical cancer", (a). From the central core, we reconstructed a PPI network (b), this network expanded and used to obtain the overrepresented GO's and pathways. Finally, we conducted an analysis of the transcription factors contained in the extended network using ChIP-Seq analysis of the ENCODE project (c).

Protein extracts of the six cancer cell lines: two cell lines which are positive for infection with HPV type 18 (HeLa and CaLo), two positive for HPV type 16 (SiHa and CasKi), and two HPV negative (ViBo and C-33A); and HaCaT, a spontaneously immortalized keratinocyte cell line we used as control, were obtained and analyzed by 2D SDS-PAGE (Figure 1). In our opinion, this experimental design permitted the identification of proteins that are common to the neoplastic phenotype regardless of the presence of intrinsic variations, such as a virus. We set the HaCaT cell line as a control because it is of epithelial origin and has an extended replicative potential, which allows us to distinguish between the biological events of transformation and immortality. Also, it has been widely used as a control in several studies of cervical cancer [14].

Three independent analyses were performed and used for protein profiling. For each sample, 2-D gel electrophoresis was carried out in a pH range of 3 to 10. The Coomassie-stained gels were acquired with densitometer GS-800 (Bio-Rad) and analyzed using PD-Quest software v 8.0.1. On average, more than 1,000 protein spots were revealed in each 2-DE gel. The quantity of each spot in a gel was normalized as a percentage of the total quantity in the map, according to its optical density (OD) value. We focused on the electrophoretic entities which were present in all six cancer cell lines but did not appear in the HaCaT control, or those which changed consistently and significantly (more than 2-fold). We call this group of 127 proteins, the "central core of cervical cancer". Among the 127 spots analyzed, 98 proteins were identified based on their tryptic peptide masses using the MALDI-TOF equipment, resulting in 66 unique proteins. (Table 1).

**Table 1:** Proteins identified as members of the "central core of cervical cancer".

Accession number	Name	Score	Expect	Searched-Matched	Coverage
GRP78_HUMAN	78 kDa glucose-regulated protein	391	1.60E-035	91 - 46	58%
ENOA_HUMAN	Alpha-enolase	327	4.00E-029	82 - 34	82%
GRP78_HUMAN	79 kDa glucose-regulated protein	302	1.30E-026	64 - 32	47%
HSP7C_HUMAN	Heat shock cognate 71 kDa protein	294	8.10E-026	70 - 39	48%
VIME_HUMAN	Vimentin	290	2.00E-025	64 - 33	63%
ENOA_HUMAN	Alpha-enolase	286	5.10E-025	82 - 34	82%
PDIA1_HUMAN	Protein disulfide-isomerase	252	1.30E-021	77 - 30	55%
ANXA2_HUMAN	Annexin A2	249	2.60E-021	65 - 31	72%
TBB5_HUMAN	Tubulin beta chain	247	4.00E-021	73 - 32	62%
ANXA4_HUMAN	Annexin A4	233	1.00E-019	50 - 25	59%
EFTU_HUMAN	Elongation factor Tu, mitochondrial	217	4.00E-018	44 - 22	52%
TPIS_HUMAN	Triosephosphate isomerase	206	5.10E-017	42 - 17	69%
VIME_HUMAN	Vimentin	206	5.10E-017	62 - 23	50%
PGAM1_HUMAN	Phosphoglycerate mutase 1	205	2.10E-016	41 - 18	58%
ATPB_HUMAN	ATP synthase subunit beta, mitochondrial	193	1.00E-015	84 - 44	72%
ACTG_HUMAN	Actin, cytoplasmic 2	192	1.30E-014	50 - 20	48%
PUR9_HUMAN	Bifunctional purine biosynthesis protein PURH	191	1.60E-015	49 - 22	45%
KCRB_HUMAN	Creatine kinase B-type	178	3.20E-014	50 - 19	48%
EF2_HUMAN	Elongation factor 2	154	8.10E-012	67 - 26	33%
HYOU1_HUMAN	Hypoxia up-regulated protein 1	154	8.10E-012	38 - 19	22%
KCRB_HUMAN	Creatine kinase B-type	154	8.10E-012	50 - 19	48%
PPIA_HUMAN	Peptidyl-prolyl cis-trans isomerase A	150	2.00E-011	45 - 16	81%
GELS_HUMAN	Gelsolin	147	4.00E-011	46 - 19	30%
VINC_HUMAN	Vinculin	145	6.40E-011	36-19	20%
WDR1_HUMAN	WD repeat-containing protein 1	144	8.10E-011	46 - 16	31%
EZRI_HUMAN	Ezrin	131	1.60E-009	60 - 26	34%
HSP71_HUMAN	Heat shock 70 kDa protein 1A/1B	129	2.60E-009	42 - 21	33%
TBA1C_HUMAN	Tubulin alpha-1C chain	127	4.00E-009	45 - 13	37%
PRDX1_HUMAN	Peroxiredoxin-1	126	5.10E-009	53 - 18	58%
PDIA3_HUMAN	Protein disulfide-isomerase A3	125	6.40E-009	47 - 15	28%
GRP75_HUMAN	Stress-70 protein, mitochondrial	123	1.00E-008	81 - 34	43%
DHSA_HUMAN	Succinate dehydrogenase flavoprotein subunit	122	3.30E-007	39 - 17	21%
RSSA_HUMAN	40S ribosomal protein SA	121	1.60E-008	37 - 11	34%
HS90B_HUMAN	Heat shock protein HSP 90-beta	119	2.6e-08	44 - 19	27%
ESTD_HUMAN	S-formylglutathione hydrolase	114	8.10E-008	25 - 11	41%
TKT_HUMAN	Transketolase	114	8.10E-008	52 - 19	32%
LDHB_HUMAN	L-lactate dehydrogenase B chain	112	1.30E-007	43 - 20	36%
AL1A1_HUMAN	Retinal dehydrogenase 1	110	2.00E-007	58 - 19	34%
IPYR_HUMAN	Inorganic pyrophosphatase	110	2.00E-007	22 - 9	35%
DDX3X_HUMAN	ATP-dependent RNA helicase DDX3X	108	3.20E-007	23 - 13	20%
DHE3_HUMAN	Glutamate dehydrogenase 1, mitochondrial	107	4.00E-007	38 - 13	29%
G3P_HUMAN	Glyceraldehyde-3-phosphate dehydrogenase	107	4.00E-007	55 - 13	42%
ACON_HUMAN	Aconitate hydratase, mitochondrial	105	6.40E-007	54 - 23	30%
ML12A_HUMAN	Myosin regulatory light chain 12A	105	6.40E-007	32 - 11	63%
RIR1_HUMAN	Ribonucleoside-diphosphate reductase large subunit	101	1.60E-006	28 - 15	18%
EF2_HUMAN	Elongation factor 2	99	2.60E-006	60-20	29%
QCR1_HUMAN	Cytochrome b-c1 complex subunit 1, mitochondrial	98	3.30E-006	45 - 15	29%
CNN2_HUMAN	Calponin-2	96	4.50E-006	51 - 13	48%
PCBP1_HUMAN	Poly(rC)-binding protein 1	94	8.30E-006	39 - 14	44%
IMMT_HUMAN	Mitochondrial inner membrane protein	92	1.30E-005	34 - 13	20%
CAPG_HUMAN	Macrophage capping protein	91	1.80E-005	41 - 11	30%
TCTP_HUMAN	Translationally-controlled tumor protein	91	1.80E-005	19 - 8	40%
PSB4_HUMAN	Proteasome subunit beta type-4	89	1.70E-003	20 - 7	34%
TERA_HUMAN	Transitional endoplasmic reticulum ATPase	88	3.10E-005	49 - 15	21%
K2C1_HUMAN	Keratin, type II cytoskeletal 1	87	3.70E-005	46 - 13	29%
LEG1_HUMAN	Galectin-1	84	7.70E-005	41 - 8	51%
1433Z_HUMAN	14-3-3 protein zeta/delta	80	2.30E-004	43 - 14	54%
LMNA_HUMAN	Prelamin-A/C	78	3.40E-004	37 - 11	22%
HNRPL_HUMAN	Heterogeneous nuclear ribonucleoprotein L	77	4.10E-004	27 - 13	16%
TCPH_HUMAN	T-complex protein 1 subunit eta	77	4.00E-004	36 - 15	30%
OSBL8_HUMAN	Oxysterol-binding protein-related protein 8	73	1.10E-003	56 - 13	21%
TCPE_HUMAN	T-complex protein 1 subunit epsilon	71	1.70E-003	30 - 11	23%
HYOU1_HUMAN	Hypoxia up-regulated protein 1	70	2.20E-003	28 - 13	14%
PSA5_HUMAN	Proteasome subunit alpha type-5	69	2.70E-003	17 - 6	25%
FOXP3_HUMAN	Forkhead box protein P3	68	3.40E-003	41 - 12	34%

The entry name corresponds to the UniProt database, the score and expect value were obtained from the search engine Mascot. The expect value is the number of matches with equal or better scores that are expected to occur by chance alone. Score as -10*LOG_10_(P), where P is the absolute probability. The lower the expectation value, the more significant the score.

Since this study pointed to a "central core of cervical cancer", it was necessary to understand the nature of the proteins involved in the process. To this end, 66 proteins were classified via GO (Gene Ontology) using QuickGo of EMBL-EBI database [15]. The identities of the proteins of the central core confirm that these are important in cancer because several studies have linked their aberrant expression to the neoplastic phenotype. With reference to their assigned functions, these proteins can be divided into at least three groups:

The first group includes proteins related to cell migration and metastasis, like anexin 2 that plays a crucial role in establishing metastasis of prostate cancer (PCa) by regulating the adhesion and migration of PCa to osteoblasts and endothelial cells [16]; protein disulfide-isomerase which is strongly expressed by invasive glioma cells, and its inhibition led to reduced glioma cell migration and invasion [17]; vimentin, an intermediate filament cytoskeletal protein and a marker of Epithelial Mesenchymal Transition (EMT), contributes to invasion and metastasis in prostate cancer [18]; ezrin is required for invasion and metastasis of mammary carcinoma [19]; and finally vinculin, which facilitates contractile force generation, enhancing cell invasion [20]. This group of proteins could be a sign of invasion and metastasis in cervical cancer, similar to their role in other cancer types.

In a second group, we placed proteins related to evasion of apoptosis: GRP78, HSP71, HSP7C, HS90B and GRP75. These proteins are activated as part of the Unfolded Proteins Response (UPR), which has been largely related to survival, cell proliferation and angiogenesis [21]. Likewise, it has been shown that overexpression of GRP78 is sufficient to confer apoptosis resistance in at least two cell types, regardless of its function within the UPR [22]. We believe these proteins could be a link between different hallmarks of cancer, like apoptosis evasion, angiogenesis and proliferation.

In a third group, we find proteins involved in or associated with central metabolism like glyceraldehyde 3 phosphate dehydrogenase, phosphoglycerate mutase 1, enolase A, triosephosphate isomerase and L-lactate dehydrogenase B. We consider that the expression of this group of proteins could be due to the cancerous cells' need to divide their incoming nutrients between energy production and macromolecular biosynthesis to support cell growth and DNA replication. This increase in cell metabolism has been described in cancer cells, which exhibit a much higher intake of glucose than normal cells, as well as increased rates of glycolysis and lactate production even in the presence of oxygen [23-26].

Another protein detected by us as a member of the "central core of cervical cancer" was galectin-1. Remarkably, very recently it was reported that cells can stimulate tumor angiogenesis by secretion of this protein, and it was previously found in the vasculature of many human tumors, including colon, head and neck, lung, prostate, and oral cancers [27,28]. All of the proteins identified as members of the "central core of cervical cancer" are summarized in Table 1.

### 14-3-3ζ is a Hub in our protein-protein interaction network

**Figure 2:**
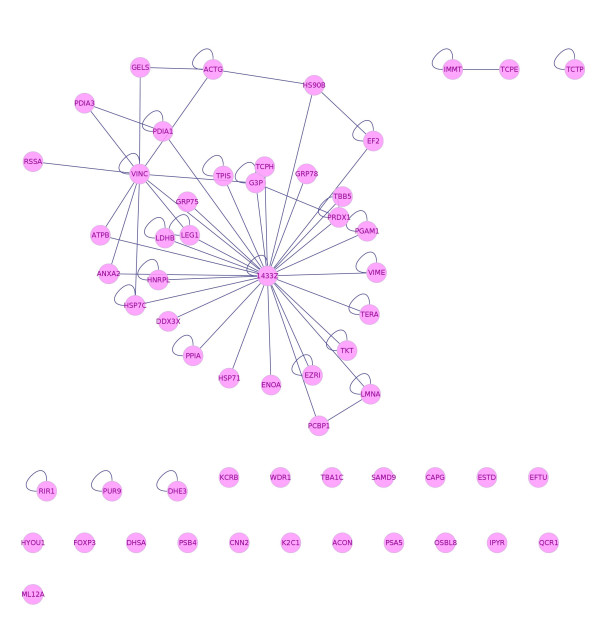
**Network Reconstruction**. Network of differentially expressed proteins in cervical cancer cell-lines compared to a non-cancerous control. The connections between proteins represent experimentally verified protein-protein interactions between 33 out of the 66 proteins identified in this study. The proteins not included in this network do not have experimentally proven interactions. The network was constructed using Cytoscape and the plugin Bisogenet.

We performed a theoretical interactome (Figure 2) using the Cytoscape plugin, Bisogenet, to add the experimentally verified interactions between the 66 identified proteins. This plugin employs the SysBiomics database which integrates information from the INTACT [29], BIOGRID [30], MINT [31], DIP [32], BIND [33] and HPRD [34] databases.

**Figure 3:**
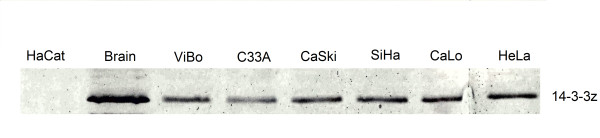
**Overexpression of 14-3-3ζ**. The expression levels of 14-3-3ζ in the six cervical cancer cell lines were assessed by western blot analysis. Whole brain extract was used a positive control, because 14-3-3ζ has a strong expression in this tissue. α-Tubulin was used as a loading control (Data not shown).

Even though we identified 66 members of the central core of cervical cancer, we were able to integrate into a network only half of them because of a lack of experimental evidence supporting PPIs. Notably, through a topological analysis carried out on this network, we found that the 14-3-3ζ protein features as a highly interconnected node (Figure 2). Additionally, western blot analysis confirmed that all cervical cancer cell lines express high protein levels of 14-3-3ζ. In contrast, no blot of this protein was found in our control (Figure 3).

Afterwards, we expanded the PPI network as a way to integrate all the proteins of the central core, in order to obtain a coherent output which could be related in its entirety to the neoplastic phenotype. It also served the purpose of broadening our perspective and strengthening the statistical significance of our results.

**Figure 4:**
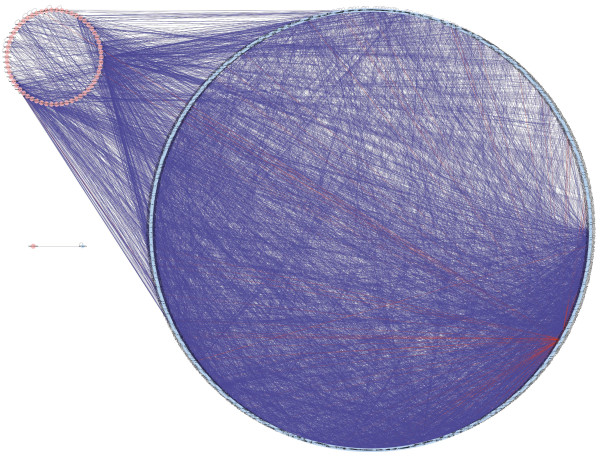
**Network Extension**. Expanded network taking identified proteins as bait. Proteins belonging to our core of differentially expressed proteins were used as bait to fish protein-protein experimental interactions by adding neighbors of input nodes to a distance of up to one protein. This resulted in a network of 1,321 nodes and 9,666 edges. The network was expanded using Cytoscape and the plugin Bisogenet. Pink nodes represent the bait proteins, while the blue nodes correspond to the proteins that were included as a result of the network expansion.

The network expansion was performed considering the experimentally obtained proteins as bait; the expansion criterion was to include proteins that have a direct experimental interaction in the databases, and were connected via a shortest path of 1 to our proteins. This resulted in a network of 1,321 nodes and 9,666 edges (Figure 4).

We considered that the network expansion would allow us to place the entities belonging to our central core in a broader context, specially because the amount of proteins that were identified represent a small fraction of all the entities responsible for the phenotype. We also expected to observe the same topological phenomena in the expanded network as we did in the network composed solely of cervical cancer-specific proteins.

A connectivity distribution analysis allowed us to identify those proteins that highly interact with others, defined as the hubs of the expanded network. This pointed once again to the multifunctional regulator of cell signal transduction and adaptor protein, 14-3-3ζ, followed by 14-3-3γ with less than half the number of interactions. Also, we calculated the betweenness of all the proteins in the network, because it is a feature which allows us to explore the connection between local network structure and global network topology, besides representing a measure of a node's influence over information transfer [35,36]. The proteins with the highest betweenness were 14-3-3ζ, vimentin and vinculin, part of our central core of cervical cancer proteins, which is consistent with their role as bait proteins. However, there were also non-bait nodes that featured among the proteins with highest betweenness, such as ubiquitin and 14-3-3γ; thus, supporting the notion that the betweenness metric, though biased in this case, is still a valid metric of essentiality and robustness.

The enrichment analysis of gene ontology level 3 category of biological processes shows 16 overrepresented GOs (Table 2); the enriched KEGG pathway-based analysis shows 16 significant pathways (Table 3). Although both enrichments are of great value for the analysis of the biological significance of our proteins, these have the limitation of considering the same protein or set of proteins as part of different, and sometimes unrelated, processes or pathways (Table 3). Consequently, results should be carefully assessed before making further interpretations.

**Table 2:** Enriched gene ontology level 3 categories of biological processes.

	*gene ontology term*	*set size*	*candidates contained*	*p-value*	*q-value*
GO:0006986	response to unfolded protein	73	8	3.11E-009	7.03E-007
GO:0070841	inclusion body assembly	13	4	4.26E-007	4.82E-005
GO:0006091	generation of precursor metabolites and energy	363	11	2.02E-006	1.52E-004
GO:0032507	maintenance of protein location in cell	80	6	3.14E-006	1.77E-004
GO:0016052	carbohydrate catabolic process	132	7	4.67E-006	1.93E-004
GO:0051651	maintenance of location in cell	87	6	5.13E-006	1.93E-004
GO:0012501	programmed cell death	1338	20	7.18E-006	2.32E-004
GO:0010941	regulation of cell death	1136	18	1.06E-005	2.99E-004
GO:0046907	intracellular transport	855	15	2.08E-005	5.21E-004
GO:0002349	histamine production involved in inflammatory response	2	2	2.56E-005	5.25E-004
GO:0043455	regulation of secondary metabolic process	2	2	2.56E-005	5.25E-004
GO:0002443	leukocyte mediated immunity	184	7	4.05E-005	7.64E-004
GO:0044248	cellular catabolic process	1028	16	4.56E-005	7.92E-004
GO:0050777	negative regulation of immune response	40	4	4.91E-005	7.93E-004
GO:0070727	cellular macromolecule localization	610	12	5.29E-005	7.98E-004
GO:0051235	maintenance of location	133	6	5.79E-005	8.18E-004

Set size refers to the number of entities that have a Uniprot ID as stated in the corresponding GO category at the ConsensusPathDB site. The number of candidates contained refers to amount of proteins identified in this study that appear as part of the GO category. P-values are calculated according to a hypergeometric test; q-values represent p-values corrected for multiple testing using the false discovery rate method.

**Table 3:** Enriched KEGG pathway-based sets.

*pathway name*	*set size*	*candidates contained*	*p-value*	*q-value*	*pathway source*
Small cell lung cancer	84	37	8.67E-009	1.43E-006	KEGG
Protein processing in endoplasmic reticulur	164	57	4.21E-008	3.47E-006	KEGG
Pathways in cancer	325	94	8.81E-008	4.05E-006	KEGG
Apoptosis	87	36	9.82E-008	4.05E-006	KEGG
Neurotrophin signaling pathway	125	46	1.24E-007	4.10E-006	KEGG
T cell receptor signaling pathway	108	41	2.25E-007	6.19E-006	KEGG
Focal adhesion	199	62	9.45E-007	2.23E-005	KEGG
Prostate cancer	89	33	6.11E-006	1.26E-004	KEGG
Pathogenic Escherichia coli infection	54	23	1.13E-005	2.07E-004	KEGG
Endometrial cancer	52	22	1.99E-005	3.28E-004	KEGG
Shigellosis	61	24	3.61E-005	5.42E-004	KEGG
Non-small cell lung cancer	54	22	4.03E-005	5.54E-004	KEGG
Proteasome	44	19	5.17E-005	6.56E-004	KEGG
Bacterial invasion of epithelial cells	70	26	5.59E-005	6.59E-004	KEGG
B cell receptor signaling pathway	75	27	7.62E-005	8.38E-004	KEGG
Leukocyte transendothelial migration	116	37	8.27E-005	8.53E-004	KEGG

Set size refers to the number of entities that have a Uniprot ID as stated in the corresponding KEGG pathway-based set at the ConsensusPathDB site. The number of candidates contained refers to amount of proteins which are part of the extended network and appear as part of the pathway. P-values are calculated according to a hypergeometric test; q-values represent p-values corrected for multiple testing using the false discovery rate method.

### There are common regulatory features among the proteins of the expanded network

Using the information generated by the ENCODE project, we searched among the transcription factors (TF) that had chromatin immunoprecipitation-sequencing (ChIP-Seq) data available, for overrepresented binding sites of a given TF in the genes of our expanded network (Figure 1). We evaluated for enrichment by considering binding sites that were located up to 700 bp upstream or 300 bp downstream of all the transcription start sites reported in the GENCODE database and that had a score larger than 750. We considered these 1,000 bp as the promoter sequence, because it has been reported to be the region of highest ChIP-Seq peak concentration in previous studies [37].

For the promoters of genes that belong to our expanded network, we focused again on the information provided in GENCODE. Out of the 1,321 nodes in the network we retrieved 1,431 promoters corresponding to 957 unique genes of the network. We found that the transcription factors E2F1 (p-value of 4.81E-13], TCF4 (p-value of 1.46E-12], c-Myc (p-value of 5.52E-11), Max (p-value of 9.77E-11), E2F6 (p-value of 1.17E-10) and NFKB (p-value of 5.02E-10) were the most significantly overrepresented by a hypergeometric test (Table 4). Importantly, the ChIP-Seq assays of the E2F1, c-Myc and Max transcription factors were performed on the HeLa-S3 cell line http://genome.ucsc.edu/. The fact that these experiments were performed in one of the cell lines we are investigating gives this bioinformatic analysis additional validity, because the cellular context is unlikely to be significantly different.

**Table 4:** Significantly overrepresented transcription factors identified by ENCODE ChIP-Seq peaks based on the GENCODE Database

*Transcription Factor*	*cell line*	*tissue of origin*	*peaks near TSS of the gene of the extended network*	*peaks near TSS of all the* *GENCODE genes*	*p-value*
E2F1	HeLa-S3	cervical	187	1445	4.81E-013
TCF4	HCT-116	colorectal	244	2059	1.46E-012
Pol2	HeLa-S3	cervical	312	2878	3.93E-011
c-Myc	HeLa-S3	cervical	123	880	5.52E-011
Max	HeLa-S3	cervical	161	1266	9.77E-011
E2F6	k562	Leukemia	265	2379	1.17E-010
NFKB	GM12878	Lymphoblastoid	111	794	5.02E-010

The peaks were mapped to regions corresponding to the promoter sequences as considered for this study (-700, 300 bp), based upon transcription start sites (TSS) annotated by GENCODE. Peaks near TSS of the genes of the extended network and all GENCODE genes refers to the amount of peaks found in the promoter region. P-values were calculated by a hypergeometric test. Sample size was considered as the amount of genes that corresponded to the proteins of the extended network. Population size was the number of annotated GENCODE entries that had a complete status and were separated by a distance of up to 500 base pairs.

Also, it is noteworthy to mention that we performed a similar analysis, but using the promoter regions as reported in the Transcriptional Regulatory Element Database (TRED] [38] and employing the SwitchGear Database for the acquisition of the transcription start sites. Using this approach, we obtained a similar enrichment (Table 5).

**Table 5:** Significantly overrepresented transcription factors identified by ENCODE ChIP-Seq peaks based on the TRED Database

*Transcription Factor*	*cell line*	*tissue of origin*	*peaks near TSS of the genes of the extended network*	*peaks near TSS reported on SwitchGear*	*p-value*
Pol2	HeLa-S3	cervical	608	7766	2.15E-067
TCF4	HCT-116	colorectal	455	5543	1.93E-050
E2F6	k562	Leukemia	488	6343	1.12E-046
Max	HeLa-S3	cervical	313	3375	2.90E-042
c-Myc	HeLa-S3	cervical	250	2395	1.88E-041
NFKB	GM12878	Lymphoblastoid	195	1802	6.78E-034
E2F1-HA	HeLa-S3	cervical	315	4272	7.57E-024

ChIP-Seq peaks mapped to regions corresponding to the promoter sequences as considered for this study (-700, 300 bp), based upon transcription start sites (TSS) annotated by SwitchGear. Peaks near TSS of the genes of the extended network and reported on SwitchGear refers to the amount of peaks found in the corresponding promoter regions. P-values were calculated by a hypergeometric test. Sample size was considered as the amount of genes that corresponded to the proteins of the extended network. Population size was the number of reported TSS reported on SwitchGear with a score greater than 20.

Among the overrepresented TFs, we consider that the presence of c-Myc is remarkable because it is in agreement to what is currently known about cancer cells in general. Also, c-Myc's primary binding partner for gene activation is Max, so that the presence of this protein is complementary to c-Myc's activity. The mechanisms that make of c-Myc a very powerful oncogene are not very clear; however, there is evidence that indicates that the activation of c-Myc correlates with approximately 70% of human cancers [39].

The first possible explanation for c-Myc's prevalence in cancer is that the genes it induces represent the primary response of nearly all signal transduction pathways known to be involved in cancer [39]. Likewise, Nilsson *et al*, [39] have suggested that a loss in c-Myc's ability to induce apoptosis can convert c-Myc into a pure promoter of cell growth and transformation. Concordantly, the abrogation of p53 function, which is the protein responsible for executing c-Myc's apoptotic response, has been substantiated in all HPV positive cell lines, as well as the HPV negative cell line, C33-A [40].

The last factor, E2F1, controls cell-cycle progression and DNA replication and it has been shown to be induced by c-Myc's activity [41]. E2F1 has also been implicated in c-Myc mediated down regulation of p27^KIP1^, which is a cyclin-dependent kinase inhibitor [42]. Finally, polymerase 2 featured as the DNA-binding protein with the most significant p-value, thereby providing us with a notion of the genes of the network that are being transcribed (Tables 4 and 5).

## Discussion

We performed a comprehensive analysis that stems from data obtained by proteomic techniques, using perspectives of biological networks and the annotated data of the human genome and large-scale sequencing techniques available to the public as a tool for building models to infer biological behaviors (Figure 1).

As defined in this study, the "central core of cervical cancer" does not describe the immortalization process directly; rather, it represents processes involved in maintaining a viable and proliferating cell. Table 2 presents the biological processes that are enriched in our core set of proteins and as can be seen, these are suggestive of a malignant phenotype, where the main GOs are those related to UPR, metabolism, catabolic processes, maintenance of cell-location and cell death. These observations are consistent with our initial conjectures, since we considered that using the HaCaT cell line as a control would allow us to distinguish between immortalization and transformation, because HaCaT is an immortalized cell line with a stable kariotype and phenotype, which is non-tumorigenic.

One of the studies that uses the HaCaT cell line as a control was performed by Choi, *et al*.[14]. The main difference between the latter study and our work, is that they used cervix biopsies; this implies that there are important differences in the intracellular and extracellular contexts of the biological model. Likewise, even though the methodology they utilized only considers samples with the same pathologic diagnosis, it is likely that there is still an important amount of heterogeneity in their samples due to inter-individual differences. As expected, we only found one common protein among the results of both studies: Vimentin. However, they were able to identify a number of proteins that strengthen our findings such as Amy-1, which stimulates c-Myc's E-box dependent transactivation activity [43]; and Miz-1, which must be inhibited in order for c-Myc to mediate apoptosis [44]. Another result that supports our findings is that they observed a downregulation of 14-3-3σ, which plays an opposite role in cell growth, compared to 14-3-3ζ [45]. Finally, they also found a clear overexpression of chaperone proteins, different from the ones in our central core.

The acquisition of the reported interactions between the identified proteins provided a first insight of the key elements in the neoplastic dynamics, and became the meter that allowed us to judge the validity of later analysis, by seeking an overall agreement between the original and the extended network. It is noteworthy that for the small network the highest interconnected node was 14-3-3ζ, and this was not lost when we expanded the network. Also, both networks are enriched in similar processes.

When analyzing the extended network it is apparent that 6 of the members of the 14-3-3 protein family (ζ, γ, β, τ, σ and ε) feature among the highest interconnected nodes. This is consistent with the biological role of the members of this protein family as signal transduction molecules [46]. The identification of 14-3-3ζ and its role as a hub and as a bottleneck in the PPI network is particularly relevant because it is known to be involved in three important cellular processes: cell cycle regulation, signal transduction and regulation of apoptosis [47-50].

**Figure 5:**
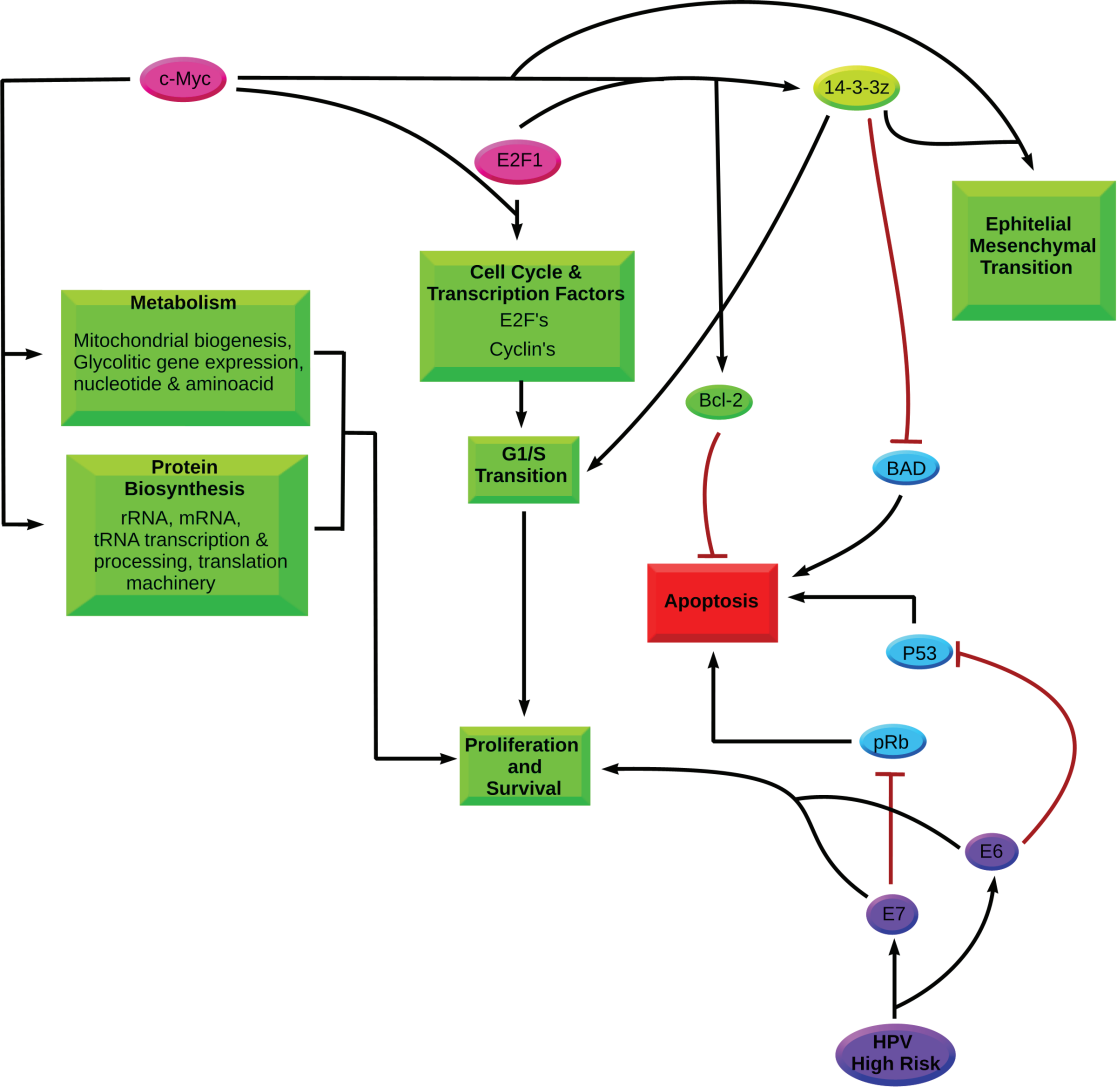
**Model of Action of c-Myc, E2F1 and 14-3-3ζ**. Model of the downstream events product of the overexpression and/or amplification of c-Myc, and its collaboration with the transcription factor E2F1. c-Myc promotes the expression of proteins that lead to survival and proliferation through processes such as metabolism, protein biosynthesis and transcription factors. Likewise, it enables the expression of proteins involved in epithelial mesenchymal transition. E2F1 and c-Myc work together to promote the expression of Cyclins and E2F factors that boost the transition between G1 and S phases of mitosis, as well as the expression of 14-3-3ζ and Bcl-2. Bcl-2 is an antiapoptotic protein that prevents the release of cytochrome c. The overexpression of 14-3-3ζ results in instability and degradation of p53, increased cell proliferation, and cytoplasmic sequestration of BAD with what brings drastic decrease of apoptosis.

Furthermore, we noticed that the 14-3-3ζ protein, which features as the highest interconnected node of both our networks, did not posses any ChIP-Seq peak near the transcription start site of its encoding gene. Upon more careful inspection throughout the entire length of the gene, we noticed that there appears to be a regulatory region inside the transcript. Importantly, we found binding sites of c-Myc, Max, E2F1 and Pol2 very close to each other. This has lead us to believe that one of the mechanisms that lead to 14-3-3ζ's overexpression is mediated by c-Myc and E2F1 (Figure 5).

It was previously reported by Jonsson and Bates [13] that cancer proteins exhibit a significantly different network topology when compared to proteins unrelated to cancer; as well as a higher ratio of promiscuous structural domains. This is in agreement with what is known about the members of the 14-3-3 protein family, which have been described as signal integrators, amplifying strong signals and filtering out weaker conflicting ones to achieve a meaningful, coordinate biological output, such as cell death or survival [51].

Also, there is strong evidence that the overexpression of 14-3-3ζ promotes p53 degradation by the proteosomal route [52], which is increased by the action of E6 in HPV-infected cells [7]. An enhanced turn-over rate of the p53 protein and the pleiotropic effects consequence of the increased number of 14-3-3ζ proteins, which are involved in a large number of cellular processes, could be the reason why not all infections develop into cancer and most of them are subclinical: c-Myc could be activating 14-3-3ζ in a differential manner. Therefore, we consider that we posses sufficient evidence to suggest the role of 14-3-3ζ in cervical cancer cell lines as the determining factor that can ultimately dictate the fate of a cell, regardless of it being infected with HPV; and cause it to undergo cell cycle deregulation onto malignant transformation (Figure 5).

Likewise, when we performed a biological pathway enrichment, which we consider the best way to perform a functional analysis, we found consistent results. To do this, we used the ConsensusPathDB site to find overrepresented KEGG pathways in the proteins of our extended network. The enrichment analysis showed an overrepresentation of survival factors of apoptosis pathways and focal adhesion that is related to invasion and metastasis, two of the most important and well-documented hallmarks of cancer; as well as various established cancer pathways like small cell lung cancer, prostate cancer, endometrial cancer and pathways in cancer (Table 3). This was obviously expected and helps to strengthen our analysis, indicating that the central core of cervical cancer, and its subsequent expansion proposed in the PPI interaction network, still retain those proteins that are closely related to the neoplastic process.

However, there are other pathways that do not present such a straightforward connection to cancer, such as the neurotrophin and T-cell receptor signaling pathways, bacterial infections and proteasome pathways. We can only speculate about their meaning, as the cellular stress, chronic inflammation, turnover of proteins, cell survival and evasion of the immune system are other features related to the progression and maintenance of tumorigenesis.

As a way to understand the underlying regulatory dynamics of the system, we also performed an enrichment analysis of transcription factor binding sites among the promoters of the genes of our extended network. We focused on the three transcription factors that complied with both of the following: those which were significantly overrepresented among the promoter regions of the genes of the proteins of our network and those in which the corresponding ChIP-Seq assays were carried out on the HeLa-S3 cell line http://genome.ucsc.edu/. The resulting transcription factors are: E2F1, c-Myc and Max. We believe that together, these transcription factors bring an overall feeling of coherence to the network because the overexpression of c-Myc is widespread in human cancers, and specifically in cervical cancer [53]. Moreover, there is evidence that E2F1 is activated by the E7 protein of HPV-16 in a pRB-independent manner [54], apart from its pRB-dependent activation, which in the case of HPV infected cells is also promoted by pRB inactivation by viral oncoprotein E7. In the context of cervical cancer, it has been reported that an overexpression of E2F1 can drive quiescent cells through G1 into S-phase of the cell cycle, ultimately leading to apoptosis or neoplastic transformation.

E2F1 has been shown to be an inhibitor of c-Myc's activator, β-catenin, by means of the Wnt pathway [55,56]. We also found a clear overrepresentation of the TCF4 transcription factor in the genes of our network. This contradicts our previous finding because TCF4 functions in the activation of the Wnt pathway, and therefore, of c-Myc. However, we had to consider that all the published works that point to E2F1 as an inhibitor of c-Myc have been performed in colorectal cancer. Likewise, the evidence of enrichment of TCF4 binding sites was performed in the HCT-116 cell line and so we do not posses information about the function of this transcription factor in cervical cancer.

It has been observed in human foreskin keratinocyte and fibroblast (HFK and HFF) cells that the genetic background of the cell type can be determinant for the outcome of altered gene expression. This is the case of HFF which cannot be immortalized by the mere addition of oncoproteins E6 and E7, unlike the HFK cells, due to a differential turnover of c-Myc [57]. Moreover, as pointed by Bernards [58] in the context of colorectal cancer pRB is more likely to be acting as an oncoprotein than as a tumor suppressor, which is clearly not the case in cervical cancer. Considering all the previous evidence, we can only infer that the proteins pRB and E2F1 are acting with opposing roles in cervical cancer, as compared to colorectal cancer.

On the other hand, there are reports of E2F1 and c-Myc overexpression in cervical cancer, which also correlate their expression with advanced states of the disease [59]. Also, genetic studies of the E2F promoters have shown that these genes are induced by c-Myc, dependent on the E box sites. Coordinately, all data point to a model where c-Myc is activated by means different from the canonical pathways and is working with E2F1 to promote the neoplastic phenotype.

The possible scenario of molecular events in cervical cancer suggested by our study portrays the evasion of apoptosis mediated in two ways, first, the sequestration of the pro-apoptotic protein BAD by 14-3-3ζ [60] and the overexpression of the anti-apoptotic protein Bcl-2 managed by the interaction of c-Myc and E2F1 [61]. Evasion of apoptosis by these routes can be supported by the interaction of viral oncoproteins E6 and E7 with tumor suppressor proteins p53 and pRb, when there is persistent infection with high-risk HPV. Simultaneously, these oncoproteins are able to promote significant increase in proliferation, and immortalize cells through the activation of hTERT [62].

The expression of E2F factors and cyclins facilitated by c-Myc, together E2F1 leads to a quick transition from the G1 to the S phase of the cell cycle, and the consequent boost in growth and cell proliferation [63].

Finally, c-Myc overexpression allows a significant increase in cell proliferation by key roads, such as protein biosynthesis, central metabolism, the expression of transcription and cell cycle factors [64] and expression of 14-3-3ζ. All of these contribute to facilitate the transition from G1 to S phase [65]. c-Myc's mediated expression of Ezrin [66] also promotes epithelial mesenchyma transition, facilitated by the overexpression of vimentin [67] (Figure 5).

## Conclusions

Our results suggest a phenotype shared by the six cervical cancer cell lines as a result of the overexpression of c-Myc, helped by E2F1, which in turn allows the overexpression of 14-3-3ζ and other proteins of the "central core of cervical cancer". This signal transduction protein has been reported in other models of cancer as being responsible for malignant transformation and the decision between life and death of cells (Figure 5).

## Methods

### Cell culture

The CaSki, HeLa, SiHa, C-33A, ViBo and CaLo cell lines were provided by the oncology laboratory of the Centro Medico Siglo XXI which belongs to the Instituto Mexicano del Seguro Social. The HaCaT cell line was donated by the Centro de Investigación Sobre Enfermedades Infecciosas, which belongs to the Instituto Nacional de Salud Pública. All cell lines were cultured in RPMI-advanced 1640 serum-free media (Gibco BRL, USA) with red phenol and antibiotic-antimycotic solution (10,000 units penicillin, 10 mg streptomycin, and 25 μg amphotericin B per mL), supplemented with 1% fetal bovine serum (Invitrogen, Carlsbad, CA) and 200 mM of GlutaMAX (Invitrogen). The cells were incubated in 5% of CO2 and humidity saturation at 37°C in culture flasks of 75 cm^2 ^(Nalge Nunc International, Rochester, NY). Cells were harvested at 70% confluence with Verseno solution (Tris base 25 mM, NaCL 136.8 mM, KCl 5.36 mM, EDTA 1 mM pH7.7) and washed 3 times in phosphate buffer saline (0.1 M sodium phosphate and 0.15 M NaCl in one liter, pH 7.2).

### Proteomic Analysis

Protein extraction and two dimensional gel electrophoresis were done as previously described [68]. Gels were dyed in colloidal coomasie [69] and scanned in a GS-800 densitometer (Bio-Rad, Hercules, CA). Digital images were analyzed and compared using the PDQuest 8.0.1 software (Bio-Rad). Each experiment was done in triplicate. Once the digital image of each gel was compared against the rest, the electrophoretic entities of interest were cut, alkylated, reduced, digested and automatically transferred to a MALDI analysis target by a Proteineer SP II and SP robot using the SPcontrol 3.1.48.0 v software (Bruker Daltonics, Bremen, Germany), with the aid of a DP Chemicals 96 gel digestion kit (Bruker Daltonics) and processed in a MALDI-TOF Autoflex (Bruker Daltonics) to obtain a mass fingerprint. We performed 100 satisfactory shots in 20 shotsteps, the peak resolution threshold was set at 1,500, the signal/noise ratio of tolerance was 6, and contaminants were not excluded. The spectrum was annotated by the flexAnalysis 1.2 v SD1 Patch 2 (Bruker Daltonics). The search engine MASCOT [70] was used to compare the fingerprints against the UNIPROT [71] release 2010_09 database with the following parameters: Taxon-Human, mass tolerance of up to 500 ppm, one miss-cleavage allowed, and as the fixed modification Carbamidomethyl and oxidation of methionine as the variable modification.

### Western blot analysis

Antibodies for immunoblotting were as follows: anti-14-3-3ζ human polyclonal (Imgenex, San Diego, CA) and anti-α-tubulin mouse monoclonal (Zymed Laboratories, South San Francisco. CA). Equal amounts of protein samples were subjected to SDS-PAGE, and transferred to a polyvinylidene difluoride membrane (Immobilon-P, Millipore Corp., Billerica, MA, USA). After blocking with 5% skimmed milk, the membrane was washed in TBS-tween 20%, and incubated with a primary antibody, followed by a AP-conjugated second antibody anti-rabitt or anti-mouse (Zymed). Blots were detected by chromogenic substrate BCIP/NBT (Zymed).

### Network Reconstruction

The network reconstruction was performed with the aid of the Cytoscape [72] Plugin, BisoGenet [73], using the identified proteins as bait nodes and adding edges with the following parameters: Organism> Homo sapiens, protein identifiers only; Data Settings>protein-protein interactions; all data sources and all experimental methods; method> By adding edges connecting input nodes and as Output> Proteins.

### Network Extension

The primary network was extended by performing the bioinformatic bait technique, using the Cytoscape Plugin Bisogenet with the expand network option and the previous parameters, except that the method used was By adding neighbors of input nodes to a distance of one. The resulting network was subjected to a centrality measure analysis with the Plugin CentiScaPe1.1 [74].

### Pathway and GO enrichment analysis

We performed an enrichment analysis of pathway-based sets of proteins considering all the nodes of our extended network. Enrichment was done employing ConsensusPathDB [75], of the Max Planck Institute for Molecular Genetics, by using the overrepresentation analysis online tool. As input, we uploaded the UNIPROT protein identifiers of all the elements of the extended network. We searched against pathways as defined by KEGG [76], with a minimal overlap with the input list of 5 and a p-value cutoff of 0.0001.

Also, employing the same website and the same analysis tool, we performed an enrichment analysis based on Gene Ontology [77] level 3 category of biological processes. For this analysis, we considered only the identified core proteins and set the p-value cutoff on 0.00001.

### Promoter Analysis

To help us uncover some of the regulatory dynamics underlying cervical cancer we downloaded the Gencode Genes-ENCODE Gencode Gene Annotations table (wgEncodeGencodeManual V3) [78,79] from the University of California Santa Cruz Genome Browser website [80]. From this table we retrieved the transcription start site of all GENCODE genes with a score greater than 750, as well as its associated ENSEMBL Transcript ID and gene name. Because some transcripts present several nearby start sites, we also set a filter that only considers start sites located at a distance greater than 500 bp, this reduced the amount of entries considered to 18,534. Promoter sequences were defined as 700 bp upstream and 300 bp downstream of the transcription start site. The promoter locations corresponding to the genes of the proteins of our network were identified based on the ENSEMBL transcript ID as well as the gene names and synonyms stated in the UNIPROT data of those proteins.

Peak information was downloaded from the Yale transcription factor binding site (TFBS) track of the ENCODE Project [80] with a preference for the assays from the HeLa-S3 cell line. A peak was considered to be inside the promoter if the mid-point of the peak was inside the 1,000 bp reported as the promoter sequence. Hypergeometric tests were performed in order to assess the significance of our findings. Sample size was considered as the number of promoters that contain TFBS. All methodology is summarized in Figure 1.

The data associated with this manuscript may be downloaded from ProteomeCommons.org Tranche using the following hash:

ixDTEeYyZfAfXrh8HgM4rHTY0BDvNFtaU/GHBpQW/ldOSRKlStNTsd5aKw9dla

58 iAmEm8L7H8jAZ7A+TR2SNpTg+EUAAAAAAAAB8Q==

## Abbreviations

ChIP: Chromatin immunoprecipitation; GO: Gene Ontology; HPV: Human Papillomavirus; HR-HPV: High Risk Human Papillomavirus; HFF: Human foreskin fibroblast; HFK: Human foreskin keratinocyte; PCa: Prostate cancer; PPI: Protein-protein interaction; TF: Transcription factor; TFBS: Transcription factor binding site; UPR: Unfolded protein response; OD: Optical Density; EMT: Epithelial Mesenchymal Transition; KEGG: Kyoto Encyclopedia of Genes and Genomes; UniProt: Universal Protein Resource; ENCODE: Encyclopedia Of DNA Elements; ConsensusPathDB: ConsensusPath Database; EBI: European Bioinformatics Institute; EMBL: European Molecular Biology Laboratory.

## Authors' contributions

JCHA and MREG carried of the experiments, participated in its design and drafted the manuscript. ORA reviews the draft and was part of the discussion. MHO realized the mass spectrometry analysis and SEG conceived the study, participated in its design and coordination, and proof-read the manuscript. All authors read and approved the final manuscript.
